# Microbial threats and the global society.

**DOI:** 10.3201/eid0201.960110

**Published:** 1996

**Authors:** S B Levy


### Microbial Threats and the Global Society

The public health threat of microbial organisms living in what can be regarded as one large global society was the subject of a recent interactive workshop sponsored by Tufts University's Education for Public Inquiry and International Citizenship 10th anniversary celebration, held at Tufts University in Medford, Massachusetts. The participants* discussed microbes as the harbingers of disease and society as both potential victim and guardian of health. Microbial threats were identified as new, reemerging, and not yet known.

The forum examined the many unanswered questions regarding the origin and causes of infectious disease agents. The failure of traditional treatments due to antibiotic resistance and the ineffective control and continued spread of infectious agents were also discussed. Participants addressed environmental and behavior factors that foster the "amplification" and "spread" of disease organisms: bathhouses conducive to the spread of HIV infection, homelessness and crowded living promoting the spread of tuberculosis, day-care centers that are ideal environments for the spread of drug-resistant pneumococcus.

In the context of the workshop at Tufts, analyses of emerging infectious disease issues generated insights about the political and social framework within which to address these threats to health: A minority group may be particularly affected by a new or reemerging disease, as was the case, for example, of AIDS in the gay population or tuberculosis in the immigrant and homeless population. These groups become valuable resources for understanding the factors leading to the emergence or reemergence of the disease and should be the focus of public health efforts for curtailing its spread. However, as the history of AIDS demonstrates, because of political concerns, investigative efforts are often delayed or inadequate to stop the spread of the disease.

An emerging or reemerging organism, however, propagates and spreads unhindered by the social concerns of its potentially infectable host. To microorganisms, the world is a single entity without borders. Microorganisms have more freedom than we do and also more genetic flexibility. Thus, in the contest between humans and microbes, we are at a disadvantage. We can neither easily acquire resistance mechanisms *against* the organisms, nor rapidly respond to an infectious disease problem in another country. The recent difficulties in dealing with a possible plague epidemic in India are just one example. Moreover, antibiotics which have been a front-line weapon against diseases are becoming increasingly ineffective, and new antibiotics to treat and contain drug-resistant bacterial strains are not available.

Inadequate microbiologic diagnostic capability—also the result of the national and international political climate—works to the advantage of emerging microbes. During the plague outbreak in India, laboratory facilities that could confirm the diagnosis were lacking. In the United States, similar inadequacies in laboratory diagnostic capacity interfere with rapid reporting of common community-acquired infections and their susceptibility to antibiotics. If physicians promptly knew what they were treating, the need for use of an antibiotic as well as the proper kind of antibiotic would be based on data, not guesswork.

The emergence of antibiotic resistance was not factored into strategic planning by public health authorities. If it had been, perhaps conditions could have been in place to handle it, as well as AIDS, tuberculosis, and other emerging pathogens. Insurance against devastating happenings in infectious disease has never been given the attention it deserves. Such insurance would have been helpful, not just in money, but also in expertise to forfend and then cope with the calamity, like insurance for earthquake damage to structure and other unexpected disasters. Should we not consider insuring our future by putting more money and expertise into basic research, into systems for surveillance, and into ways to curtail the spread of a disease once it has emerged?

To meet the demands of increased public health activity and to implement an "insurance policy" for the future, we need to be able to *communicate* the problem to a broad audience that sometimes has little understanding of the science. To some public health officials, recognition that an infectious disease problem exists is sufficient to address the problem. However, to those not trained in the field who may be making important policy decisions, the "public safety" aspect of the problem can be emphasized. Health, like crime and traffic, should become once again a major society issue.

Requests for increasing support for surveillance, education, and research must take into account current political and social priorities and emphasize direct benefits to the U.S. population; international efforts should involve the collaboration of other countries.

Nongovernment agencies need to be enlisted in this public health effort; thus a larger portion of society will be involved in the fight against the ever-increasing threat of infectious diseases.

**Stuart B. Levy**
Center for Adaptation Genetics and Drug Resistance,
Tufts University School of Medicine, Boston
Massachusetts, USA

*Participants: Stuart B. Levy, Tufts University School of Medicine, and Ruth L. Berkelman, Centers for Disease Control and Prevention (co-conveners); Christopher Foreman, The Brookings Institute; Laurie Garrett, *Newsday*; Margaret Hamberg, New York City Department of Health; Joshua Lederberg, Rockefeller University; Jonathan Mann, Christopher Murray, Harvard School of Public Health.

